# Proteomic analysis of human follicular fluid based on the 4D label free method to identify proteins that may affect oocyte quality in hyperandrogenic PCOS patients

**DOI:** 10.3389/fendo.2025.1579469

**Published:** 2025-05-15

**Authors:** Qianqian Yin, Jianhua Zheng, Yijuan Cao, Xiaonan Yan, Hong Zhang

**Affiliations:** ^1^ Center for Reproductive Medicine, XuZhou Central Hospital, Xuzhou, Jiangsu, China; ^2^ Department of Obstetrics and Gynecology, The Second Affiliated Hospital of Soochow University, Suzhou, Jiangsu, China; ^3^ Jiangsu Key Laboratory of New Drug Research and Clinical Pharmacy, Xuzhou Medical University, Xuzhou, Jiangsu, China; ^4^ Department of Obstetrics and Gynecology, XuZhou Central Hospital, Xuzhou, Jiangsu, China

**Keywords:** polycystic ovary syndrome, hyperandrogenism, oocyte, follicular fluid, proteomics

## Abstract

**Objective:**

Proteomic analysis was conducted on human follicular fluid (FF) using the 4D label-free method to identify proteins potentially influencing oocyte quality in hyperandrogenic (HA) polycystic ovary syndrome (PCOS) patients.

**Methods:**

FF was collected from 3 different groups: HA PCOS patients, non-hyperandrogenic (NHA) PCOS patients, and controls. Protein profiles of FF from HA PCOS patients (n = 10) were constructed utilizing 4D label-free proteomics technology. Differentially expressed proteins were identified by comparing these profiles with those from NHA PCOS (n = 10) and control patients (n = 10). In addition, FF was collected from 34 HA, 33 NHA, and 23 control patients for enzyme-linked immunosorbent assay (ELISA) validation of differentially expressed proteins. Associations between the levels of differentially expressed proteins in FF and various embryonic outcome indicators were evaluated.

**Results:**

The HA group demonstrated significantly reduced normal cleavage rates, D3 available embryo rates, D3 high-quality embryo rates, available blastocyst rates, and high-quality blastocyst rates compared to the NHA and control groups (HA vs. NHA vs. Control, 88.3 vs. 93.6 vs. 94.23, 70.57 vs. 81.76 vs. 83.77, 42.49 vs. 56.39 vs. 61.83, 55.0 vs. 65.96 vs. 67.26, 27.62 vs. 45.19 vs. 44.75, respectively), with statistically significant differences (*P* < 0.05). 23 differentially expressed proteins were identified in FF profiles of the HA group relative to the control group, while 9 differentially expressed proteins were noted in comparison with the NHA group. Cross-comparison highlighted three potential target proteins: insulin-like growth factor binding protein 5 (IGFBP5), lysosomal-associated membrane protein 2 (LAMP2), and cadherin-5 (CDH5). Adjusting for age and body mass index (BMI), IGFBP5 levels in FF exhibited negative correlations with normal cleavage rate, D3 high-quality embryo rate, available blastocyst rate, and high-quality blastocyst rate (*P ≤* 0.05). Similarly, LAMP2 levels were negatively correlated with normal cleavage rate, D3 available embryo rate, D3 high-quality embryo rate, and high-quality blastocyst rate (*P* < 0.05). CDH5 levels demonstrated positive correlations with D3 high-quality embryo rate and high-quality blastocyst rate (*P* < 0.05).

**Conclusion:**

The proteins IGFBP5, LAMP2, and CDH5 may contribute to the mechanisms underlying the adverse effects of hyperandrogenism on oocyte quality in PCOS patients.

## Introduction

Polycystic ovary syndrome (PCOS) is a common endocrine metabolic disorder, which is characterized by oligo/anovulation, clinical or biochemical hyperandrogenism, and/or polycystic ovaries. It is known to affect approximately 6 to 10% of women of reproductive age (1). Although the international guidelines have stated PCOS as a risk factor for infertility only in the presence of oligo-ovulation or anovulation and recommend *in-vitro* fertilization-embryo transfer (IVF-ET) as a last solution, a high proportion of women with PCOS fail to conceive after ovulation and require hospital visits for infertility and IVF treatment 8 and 10 times more frequently, respectively than those without PCOS (1). Previous studies have demonstrated that even if patients with PCOS have undergone IVF to assist conception and can achieve abundant oocytes during the ovulation-promoting process of IVF, the quality of their oocytes is usually poor. In addition, the rates of normal fertilization of oocytes, egg cleavage, high-quality embryos, and blastocyst formation are significantly lower than those in the general control population (2). Therefore, in addition to ovulation disorders, decreased oocyte quality is likely to affect the fertility of patients with PCOS.

Hyperandrogenism, one of the three clinical features of the Rotterdam Criteria for PCOS, has been implicated in the pathophysiologic mechanisms underlying PCOS (3). The administration of exogenous androgen alone is the most commonly used method to establish animal models of PCOS (4). Previous studies have demonstrated that patients with PCOS without hyperandrogenism, similar to controls, have significantly higher pregnancy rates and live birth rates compared to those with hyperandrogenism (5). Thus, hyperandrogenic status reduces the pregnancy and live birth rates in patients with PCOS. It remains unknown if reduced pregnancy rate and live birth rate in patients with hyperandrogenic PCOS are related to their poor oocyte quality. Furthermore, specific mechanisms by which hyperandrogenism affects oocyte quality remain elusive.

Follicular fluid (FF), present in the lumen of growing follicles, constitutes an immediate microenvironment for oocyte development and maturation (6), thus reflecting the status of oocytes to a certain extent. Because it is ethically challenging to acquire normal oocytes and FF is a by-product that can be easily obtained during IVF egg retrieval, researchers usually study FF to assess the status of oocytes. Recently, researchers have investigated the pathogenesis of PCOS by comparing the differences in the protein profiles of FF from patients with and without PCOS (6–8), screened for biomarkers, and elucidated the possible pathogenesis of PCOS at the molecular protein level. However, no previous FF proteomics studies have addressed hyperandrogenic versus non-hyperandrogenic phenotypes within the PCOS population, with considerably few studies linking them to oocyte quality.

In the present study, we applied the 4D label-free proteomics technology to construct an FF protein profile of patients with hyperandrogenic PCOS and subsequently explore the possible mechanisms by which hyperandrogenism affects the oocyte quality of these patients. In addition, we identified possible targets for clinical diagnosis and treatment to improve the oocyte quality of patients with hyperandrogenic PCOS.

## 2 Materials and methods

### 2.1 Patient selection and stimulation protocol

Patients recruited for this study underwent IVF-ET at Xuzhou City Central Hospital between March 2021 and May 2024. These patients received superovulation treatment using an antagonist regimen and were subsequently fertilized via IVF. The study was approved by the Biomedical Research Ethics Review Committee of Xuzhou Central Hospital (approval number: XZXY-LK-20200331-027). All patients signed an informed consent form before participating.

Patients were diagnosed with PCOS according to the Rotterdam criteria (9). They were divided into the hyperandrogenic (HA) PCOS and non-hyperandrogenic (NHA) PCOS groups. The diagnosis of hyperandrogenism was based on clinical hyperandrogenism (modified FG score ≥ 8) or biochemical hyperandrogenism (serum total testosterone [TT] ≥ 2.6 nmol/L; or free androgen index (FAI ≥ 6.4, FAI = T [nmol/L] × 100/SHBG [nmol/L]); or free testosterone (FT) ≥ 20.82 pmol/L (9, 10); or DHE-S ≥ 10.6 µmol/L. The control group included patients undergoing IVF for tubal or male factor infertility. Exclusion criteria included age > 40 years, chromosomal abnormalities, history of recurrent miscarriages, ovarian tumors, history of surgery, history of malignancy, immune system disorders, history of more than three IVF failures, comorbidities, such as endometriosis, dysfunction of thyroid, adrenal, and pituitary, and the use of any medication affecting blood glucose, lipids, and sex hormones within 3 months. A total of 267 PCOS patients (159 HA patients and 108 NHA patients) and 203 control patients were recruited.

### 2.2 Sample collection

FF samples were collected from 10 patients with HA, 10 patients with NHA, and 10 controls for proteomic analysis. FF samples were also collected from an additional 34 HA, 33 NHA, and 23 control patients for enzyme-linked immunosorbent assay (ELISA) validation of differentially expressed proteins.

FF was collected by transvaginal ultrasound-guided aspiration on 35 to 37 h after the administration of human chorionic gonadotropin (HCG) (LiZhu Pharma; Zhuhai, China). Only clear FF samples without macroscopic blood contamination were included. After oocyte isolation, FF samples were centrifuged at 3000 rpm for 10 min to remove cells and insoluble particles. Subsequently, the supernatant was separated and stored at −80°C for further use.

### 2.3 4D label-free relative quantitative proteomics based on FF sample detection

#### 2.3.1 Mass spectrometry experimental procedures

##### 2.3.1.1 Protein extraction and digestion

Proteins were extracted from the FF of 30 patients. The SDT buffer (4% sodium dodecyl sulfate [SDS], 100 mM Tris-HCl, pH 7.6) was used for sample lysis and protein extraction. Protein quantification was performed using the bicinchoninic acid (BCA) protein assay kit (Bio-Rad, USA). Briefly, 20 µg of protein for each sample was mixed with 5× loading buffer and boiled for 5 min. Proteins were separated on a 4% to 20% SDS-PAGE gel (constant voltage: 180V, 45 min). Protein bands were visualized by Coomassie Blue R-250 staining.

An appropriate amount of protein was collected from each sample and trypsinized using the filter-aided proteome preparation (FASP) method described by Matthias Mann. The digested peptides of each sample were desalted on a C18 cartridge (Empore™ SPE Cartridges C18 [standard density], bed I.D. 7 mm, volume 3 mL, Sigma), concentrated by vacuum centrifugation and reconstituted in 40 µL of 0.1% (v/v) formic acid. The peptide content was estimated by ultraviolet (UV) light spectral density at 280 nm using an extinction coefficient of 1.1 for 0.1% (g/L) solution calculated from the abundance of tryptophan and tyrosine in vertebrate proteins.

##### 2.3.1.2 Liquid chromatography-tandem mass spectrometry (LC-MS/MS) data analysis

Liquid chromatography-mass spectrometry (LC-MS/MS) was performed on a timsTOF Pro mass spectrometer (Bruker) coupled to a Nanoelute (Bruker). The peptides were applied to a C18-reversed-phase analytical column (Thermo Scientific Easy column; 25 cm long, 75 μm inner diameter, 1.9 μm resin) in 95% buffer A (0.1% aqueous formic acid solution) connected to a linear gradient separation in buffer B (99.9% acetonitrile and 0.1% formic acid) at a flow rate of 300 nL/min. The mass spectrometer was operated in the positive ion mode. The applied electrospray voltage was 1.5 kV. Precursors and fragments were analyzed on a TOF detector with a mass range of m/z 100 to 1700. The timsTOF Pro was operated in parallel accumulation serial fragmentation (PASEF) mode; data acquisition in the PASEF mode was based on the following parameters: ion mobility coefficient (1/K0) value was set from 0.6 to 1.6 Vs cm^2^; 1 MS and 10 MS/MS PASEF scans. Active exclusion was enabled with a release time of 24 s.

#### 2.3.2 Bioinformatics analysis

The raw MS data of each sample were retrieved, identified, and quantitatively analyzed using the MaxQuant 1.6.14 software. The database was (Swissprot_Homo_sapiens_20376_20220104.fasta). Subcellular localization was predicted using the CELLO method (http://cello.life.nctu.edu.tw/). For selected genes, functional classification and enrichment analyses were performed using the ToppGene online tool (https://toppgene.cchmc.org/enrichment.jsp). The overall molecular function (MF), biological process (BP) and cellular component (CC) of the genes were analyzed using the built-in GO triple classification annotation data of ToppGene, and the Reactome data of the genes were used to analyze the regulatory pathways in which the gene clusters were mainly involved. We also used ToppGene’s built-in Reactome data to analyze the regulatory pathways in which the gene clusters are mainly involved. Finding direct and indirect interactions between target proteins based on information in the STRING (http://string-db.org/) database.

### 2.4 ELISA validation

The differential abundance of the three selected proteins was verified by ELISA using FF samples from patients in HA, NHA, and control groups. ELISA was performed on FF samples, namely, LAMP2, IGFBP5, and CDH5, according to the manufacturer’s instructions. The dilution ratios were 1:2, 1:200, and 1:1000.

### 2.5 Statistics analysis

All data were analyzed using the statistical software SPSS version 24.0 for Windows. Continuous variables are described as mean ± standard deviation ( $\bar{x}$ ± s). Differences between the groups were determined by Student’s *t*-test or one-way analysis of variance (ANOVA). Partial correlation analysis was conducted to investigate the connections between the selected protein and various embryo quality assessment indices after adjusting for age, body mass index (BMI). A statistically significant difference was defined as *p ≤ 0*.05 (two-tailed).

## 3 Results

### 3.1 Comparison of general conditions among three groups of patients

We collected data from 203 control, 108 NHA, and 159 HA patients. A comparison of baseline characteristics and ovulation promotion outcomes in the three groups is shown in **Table 1**.

**Table 1:** Comparison of clinical, hormonal and biochemical characteristics and embryonic conditions in three groups of patients.

Variables	Control (n=203)	NHA (n=108)	HA (n=159)	*P*
Age (year)	30.46 ± 3.25	29.85 ± 3.85	30.01 ± 3.73	0.277
BMI (kg/m^2^)	24.57 ± 2.67	24.19 ± 2.63	24.78 ± 3.50	0.286
AMH (ng/mLr)	4.32 ± 2.48 a	9.46 ± 4.53	10.21 ± 5.01 c	0.000
bFSH (mIU/mL)	7.66 ± 2.05 a	6.68 ± 1.39	6.56 ± 1.52 c	0.000
bLH (mIU/mL)	4.18 ± 1.63 a	5.87 ± 3.47	6.40 ± 3.81 c	0.000
mFG	0.22 ± 0.50 a	1.05 ± 1.13 b	2.30 ± 1.91 c	0.000
TT (ng/dL)	41.50 ± 13.60 a	47.41 ± 12.88 b	71.29 ± 22.06 c	0.000
SHBG (nmol/L)	55.09 ± 28.58	53.39 ± 37.36 b	32.77 ± 28.80 c	0.000
FAI	3.21 ± 1.62	3.84 ± 1.65 b	11.15 ± 7.47 c	0.000
FT (pg/mL)	1.79 ± 0.90	1.79 ± 0.70 b	2.68 ± 1.09 c	0.000
DHE-S (umol/L)	5.87 ± 1.91	5.77 ± 1.73 b	8.69 ± 3.29 c	0.000
FPG (mmol/L)	5.52 ± 0.60	5.49 ± 0.62 b	5.78 ± 1.17 c	0.004
FIN (uU/mL)	11.77 ± 6.87	13.76 ± 7.48 b	20.27 ± 13.50 c	0.000
HOMA-IR	2.99 ± 2.44	3.39 ± 1.92 b	5.44 ± 4.54 c	0.000
TCHOL (mmol/L)	4.43 ± 0.82	4.56 ± 0.85 b	4.78 ± 0.90 c	0.001
TG (mmol/L)	1.12 ± 1.11	1.25 ± 0.58 b	1.65 ± 1.37 c	0.000
HDL-c (mmol/L)	1.44 ± 0.29 a	1.34 ± 0.32	1.29 ± 0.28 c	0.000
LDL-c (mmol/L)	2.57 ± 0.74	2.74 ± 0.76	2.89 ± 0.76 c	0.001
Total amount of Gn used(IU)	2017.08 ± 612.87 a	1733.99 ± 474.81 b	1928.86 ± 675.84	0.001
number of eggs obtained	9.58 ± 4.47 a	13.64 ± 5.35	14.04 ± 5.88 c	0.000
Normal fertilization rate (%)	83.92 ± 18.32	82.50 ± 20.09	79.06 ± 19.49 c	0.101
Normal cleavage rate (%)	94.23 ± 10.18	93.60 ± 11.55 b	88.30 ± 16.23 c	0.000
D3 rate of available embryos (%)	83.77 ± 17.02	81.76 ± 16.56 b	70.57 ± 23.89 c	0.000
D3 high-quality embryo rate (%)	61.83 ± 24.63	56.39 ± 25.16 b	42.49 ± 25.51 c	0.000
Available blastocyst rate (%)	67.26 ± 23.98	65.96 ± 22.25 b	55.00 ± 28.96 c	0.000
High-quality blastocyst rate (%)	44.75 ± 24.07	45.19 ± 25.29 b	27.62 ± 24.67 c	0.000

a. Control vs NHA, *P*<0.05.

b. NHA vs HA, *P*<0.05.

c. Control vs HA, *P*<0.05.

BMI, body mass index; AMH, anti-Müllerian hormone; FSH, follicle-stimulating hormone; LH, luteinizing hormone; mFG, modified Ferriman-Gallway scores; TT, total testosterone; SHBG, sex hormone-binding globulin; FAI, free androgen index; FT, free testosterone; DHE-S, dehydroepiandrosterone sulfate; FPG, fasting plasma glucose; FIN, fasting insulin; HOMA-IR, Homeostasis Model Assessment of Insulin Resistance; TCHOL, total cholesterol; TG, triglycerides; HDL-c, high-density lipoprotein cholesterol; LDL-c, low-density lipoprotein cholesterol.

### 3.2 Quantitative FF protein profiling

**Figure 1:**
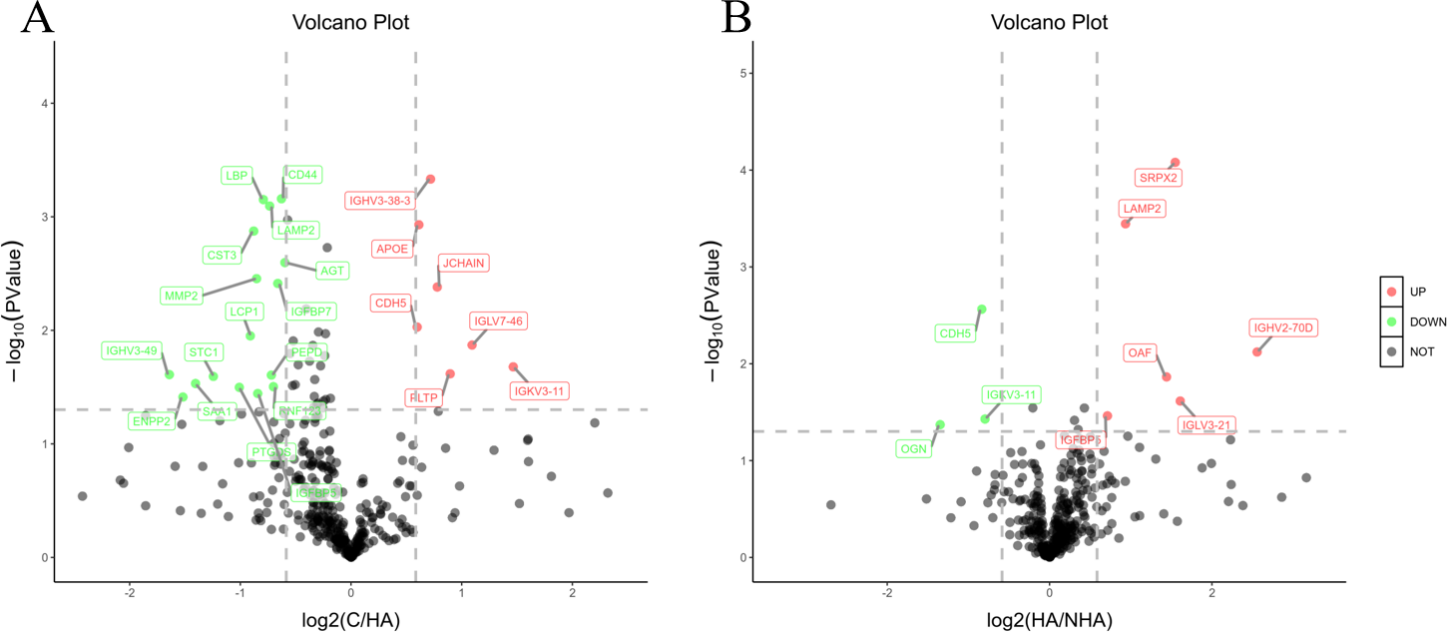
Volcano plot of differentially expressed proteins between different groups. Green points in the graph are differential proteins with down-regulated expression, red points are differential proteins with up-regulated expression, and gray points are proteins with no differential change; vertical coordinates are P-values (logarithmic with base 10), and horizontal coordinates are multiplicity of differences (logarithmic with base 2). **(A)**: C/HA (control group vs. hyperandrogenic PCOS group); **(B)**: HA/NHA (hyperandrogenic PCOS group vs. non-hyperandrogenic PCOS group).

We performed 4D label-free relative quantitative proteomic analysis on 30 samples, which identified 537 proteins, of which 435 proteins had quantitative information. A fold change (FC) value of more than 1.5-fold change and *P* < 0.05 were used as the screening criteria, and 23 differentially expressed proteins were screened between HA and control groups (**Figure 1A**), and nine differentially expressed proteins were screened between HA and NHA groups (**Figure 1B**). Information on annotation and quantification of differentially expressed proteins is listed in **Tables 2** and **3**. The subcellular localization of differentially expressed proteins was primarily concentrated in the extracellular region (HA/C: 53.33%, HA/NHA: 58.33%).

**Table 2:** Annotation and quantitative information of differentially expressed proteins in the follicular fluid of the HA and Control groups.

Protein	Gene Name	Protein Name	HA/C	*p* value
Up-regulation				
A0A0A0MS15	IGHV3-49	Immunoglobulin heavy variable 3-49	3.119	2.46E-02
Q13822	ENPP2	Ectonucleotide pyrophosphatase/phosphodiesterase family member 2	2.865	3.87E-02
P0DJI8	SAA1	Serum amyloid A-1 protein	2.648	2.94E-02
P52823	STC1	Stanniocalcin-1	2.368	2.55E-02
P41222	PTGDS	Prostaglandin-H2 D-isomerase	2.013	3.18E-02
P13796	LCP1	Plastin-2	1.879	1.12E-02
P01034	CST3	Cystatin-C	1.839	1.34E-03
P08253	MMP2	72 kDa type IV collagenase	1.806	3.51E-03
P24593	IGFBP5	Insulin-like growth factor-binding protein 5	1.793	3.60E-02
P18428	LBP	Lipopolysaccharide-binding protein	1.731	7.06E-04
P13473	LAMP2	Lysosome-associated membrane glycoprotein 2	1.664	8.04E-04
P12955	PEPD	Xaa-Pro dipeptidase	1.647	2.48E-02
Q5XPI4	RNF123	E3 ubiquitin-protein ligase RNF123	1.626	3.14E-02
Q16270	IGFBP7	Insulin-like growth factor-binding protein 7	1.581	3.87E-03
P16070	CD44	CD44 antigen	1.546	6.95E-04
P01019	AGT	Angiotensinogen	1.513	2.53E-03
Down-regulation				
P04433	IGKV3-11	Immunoglobulin kappa variable 3-11	0.362	2.10E-02
A0A075B6I9	IGLV7-46	Immunoglobulin lambda variable 7-46	0.469	1.35E-02
P55058	PLTP	Phospholipid transfer protein	0.537	2.41E-02
P01591	JCHAIN	Immunoglobulin J chain	0.583	4.16E-03
P0DTE1	IGHV3-38-3	Probable non-functional immunoglobulin heavy variable 3-38-3	0.608	4.65E-04
P02649	APOE	Apolipoprotein E	0.654	1.17E-03
P33151	CDH5	Cadherin-5	0.661	9.37E-03

**Table 3:** Annotation and quantitative information of differentially expressed proteins in the follicular fluid of the HA and NHA groups.

Protein	Gene Name	Protein Name	HA/NHA	*p* value
Up-regulation				
A0A0C4DH43	IGHV2-70D	Immunoglobulin heavy variable 2-70D	5.883	7.56E-03
P80748	IGLV3-21	Immunoglobulin lambda variable 3-21	3.052	2.43E-02
O60687	SRPX2	Sushi repeat-containing protein SRPX2	2.924	8.32E-05
Q86UD1	OAF	Out at first protein homolog	2.715	1.37E-02
P13473	LAMP2	Lysosome-associated membrane glycoprotein 2	1.913	3.59E-04
P24593	IGFBP5	Insulin-like growth factor-binding protein 5	1.639	3.45E-02
Down-regulation				
P20774	OGN	Mimecan	0.393	4.26E-02
P04433	IGKV3-11	Immunoglobulin kappa variable 3-11	0.576	3.72E-02
P33151	CDH5	Cadherin-5	0.561	2.73E-03

**Figure 2:**
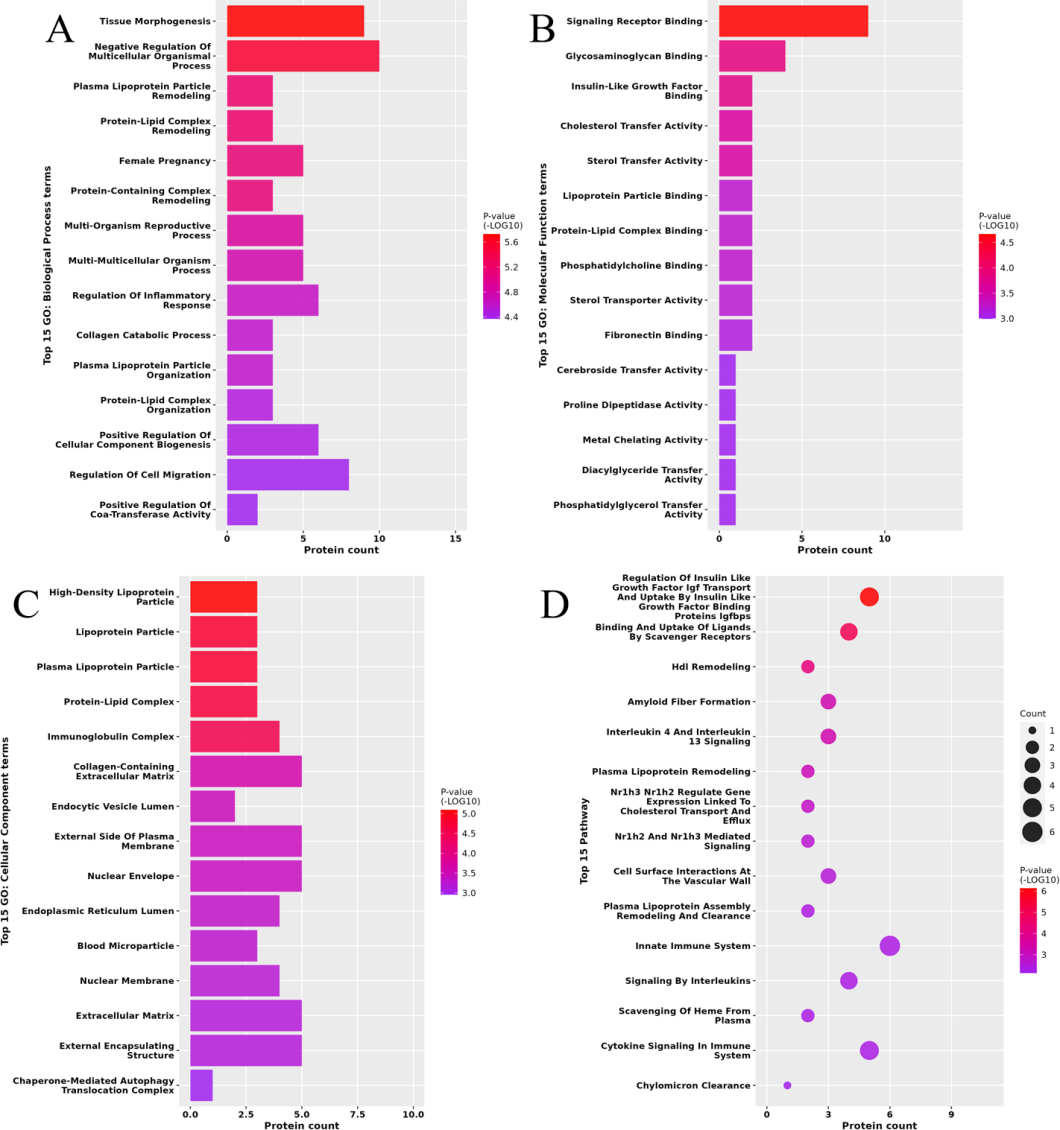
Functional enrichment of differentially expressed proteins (DEPs) in follicular fluid of hyperandrogenic (HA) and control patients. Biological process (BP) analysis **(A)**. Molecular function (MF) analysis **(B)**. Cellular component (CC) analysis **(C)**. Reactome enrichment analysis **(D)**.

GO functional annotation results demonstrated that 23 differentially expressed proteins of HA/C were primarily enriched in 2,246 GO terms. Among them, 1,876 terms were related to BP, with 855 statistically significant. Differentially expressed proteins were most enriched in biological processes such as tissue morphogenesis, regulation of multicellular biological processes, remodeling of protein–lipid complexes, female pregnancy, inflammatory response, collagen catabolic process, and cell migration (**Figure 2A**). Furthermore, 191 were related to MF, with 101 statistically significant, and the molecular functions largely corresponded to binding functions, such as signaling receptor binding, glycosaminoglycan binding, insulin-like growth factor binding, lipoprotein particle binding, and activation of cholesterol transfer, and sterol transfer (**Figure 2B**). Similarly, 179 terms were related to CC, with 70 statistically significant; differentially expressed proteins were largely implicated in the formation of different lipoprotein particles, protein–lipid complexes, immunoglobulin complexes, and several extracellular matrices (**Figure 2C**). The pathways enriched were identified by Reactome enrichment analysis (**Figure 2D**). The main pathways of enrichment were immune and metabolism-related pathways.

**Figure 3:**
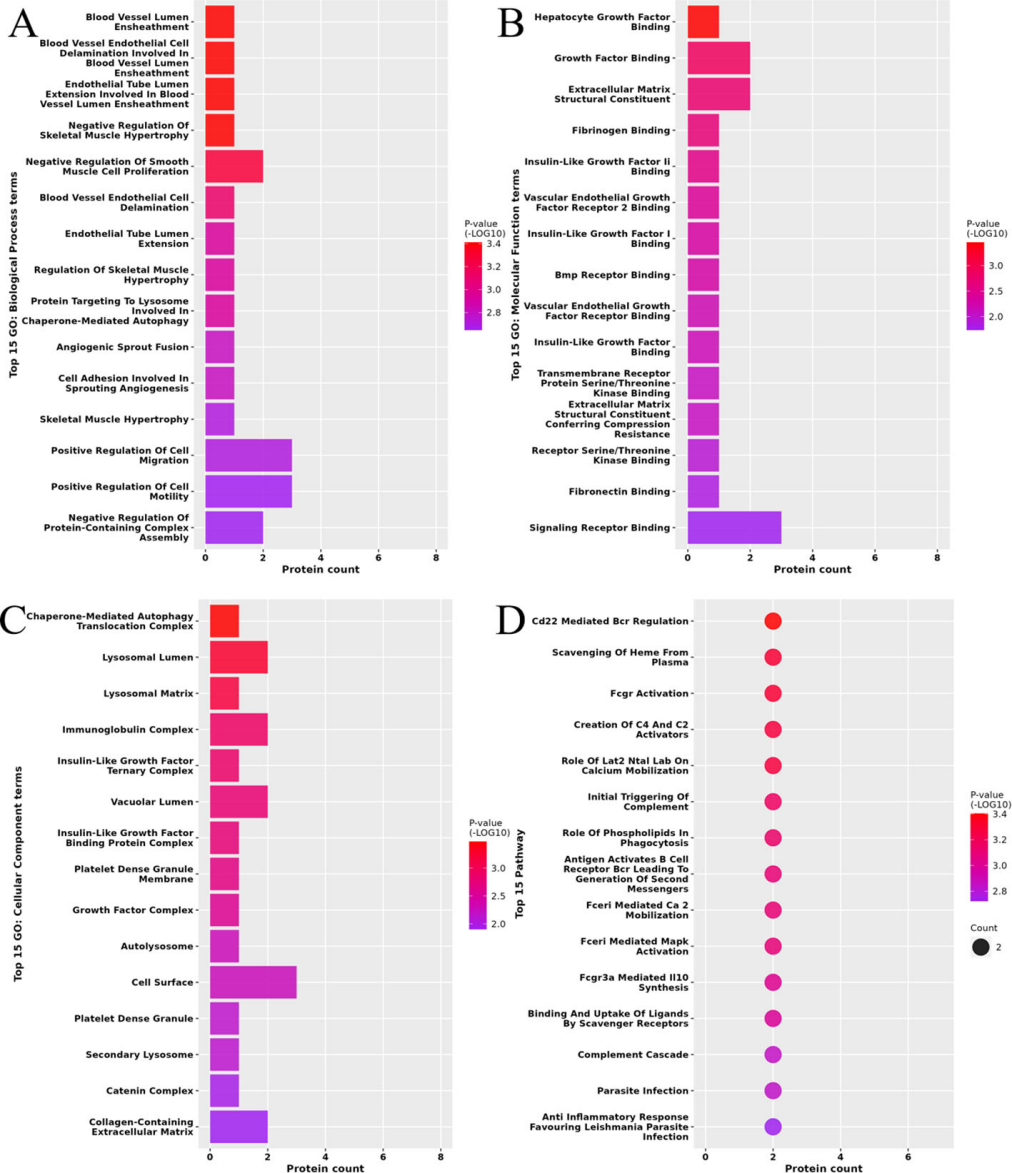
Functional enrichment of differentially expressed proteins (DEPs) in follicular fluid of hyperandrogenic (HA) and non-hyperandrogenic (NHA) PCOS patients. Biological process (BP) analysis **(A)**. Molecular function (MF) analysis **(B)**. Cellular component **(CC)** analysis **(C)**. Reactome enrichment analysis **(D)**.

HA/NHA differentially expressed proteins were majorly enriched in 506 GO terms. Among them, 395 were related to BP, with 139 statistically significant, and the differentially expressed proteins were largely involved in biological processes such as blood vessel lumen ensheathment, vascular endothelial cell stratification, endothelial tubular lumen expansion, and negative regulation of smooth muscle cell proliferation (**Figure 3A**). Furthermore, 37 were related to MF, with 19 statistically significant, and were majorly based on the binding function, including hepatocyte growth factor binding, growth factor binding, fibrinogen binding, insulin-like growth factor binding, vascular endothelial growth factor receptor binding, and BMP receptor binding function (**Figure 3B**). Next, we found that 74 were related to CC, with 32 statistically significant, and largely involved in the composition of autophagy translocation complex, autophagic vesicle, lysosome, immunoglobulin complex, insulin-like growth factor complex, and platelet dense granule membrane (**Figure 3C**). The pathways enriched were identified by Reactome enrichment analysis (**Figure 3D**). The main pathways of enriched were the complement cascade pathway and the inflammation-related pathway.

### 3.3 Validation of three differentially expressed proteins

The clinical data revealed the proteins with statistically significant differences between the HA and NHA groups, and between the HA and control groups, and those with statistically insignificant differences between the NHA and control groups were considered target proteins. Finally, three target proteins were screened, namely, LAMP2, IGFBP5, and CDH5. ELISA results are shown in **Table 4**. As demonstrated in **Table 4**, patients in the HA group exhibited notably elevated IGFBP5 and LAMP2 levels in FF, while CDH5 levels were considerably reduced compared to those in the NHA and control groups (*P* < 0.05).

**Table 4:** ELISA validation results for the three target proteins.

Variables	Control (n=23)	NHA (n=33)	HA (n=34)	*P*
Age(year)	30.70 ± 3.52	31.30 ± 3.84	30.21 ± 3.32	0.456
BMI(kg/m^2^)	23.16 ± 2.88	23.18 ± 2.95	24.22 ± 2.48	0.219
IGFBP5(ng/mL)	91.33 ± 12.89	99.00 ± 17.75 b	122.40 ± 23.13 c	0.000
LAMP2(ng/mL)	10.85 ± 5.58	12.29 ± 5.88 b	19.06 ± 6.30 c	0.000
CDH5(ug/mL)	15.63 ± 4.76	14.66 ± 4.24 b	11.01 ± 4.05 c	0.000

a. Control vs NHA, *P*<0.05.

b. NHA vs HA, *P*<0.05.

c. Control vs HA, *P*<0.05.

### 3.4 Correlation analysis between three differentially expressed proteins in follicular fluid and embryonic outcome

After correcting for age and BMI, the levels of IGFBP5 in the FF were negatively correlated with the normal cleavage rate, D3 high-quality embryo rate, available blastocyst rate, and high-quality blastocyst rate (*P ≤* 0.05). Similarly, the levels of LAMP2 in the FF were negatively correlated with the normal cleavage rate, D3 available embryo rate, D3 high-quality embryo rate, and high-quality blastocyst rate (*P* < 0.05). Furthermore, the levels of CDH5 within the FF were positively correlated with the rate of D3 high-quality embryos and the rate of high-quality blastocysts in patients (*P* < 0.05, **Table 5**).

**Table 5:** Correlation analysis of IGFBP5, LAMP2, and CDH5 levels in follicular fluid with embryonic outcome after correction for age and BMI.

Variables	IGFBP5		LAMP2		CDH5	
	r	*p*	r	*p*	r	*p*
Normal fertilization rate (%)	-0.170	0.140	-0.058	0.616	-0.010	0.934
Normal cleavage rate (%)	-0.225	0.050	-0.285	0.012	0.044	0.704
D3 rate of available embryos (%)	-0.132	0.251	-0.238	0.037	0.179	0.120
D3 High quality embryo rate (%)	-0.241	0.035	-0.305	0.007	0.368	0.001
Available blastocyst rate (%)	-0.260	0.022	-0.197	0.085	0.158	0.170
High quality blastocyst rate (%)	-0.451	0.000	-0.337	0.003	0.289	0.011

### 3.5 Interactions between the three target proteins

**Figure 4:**
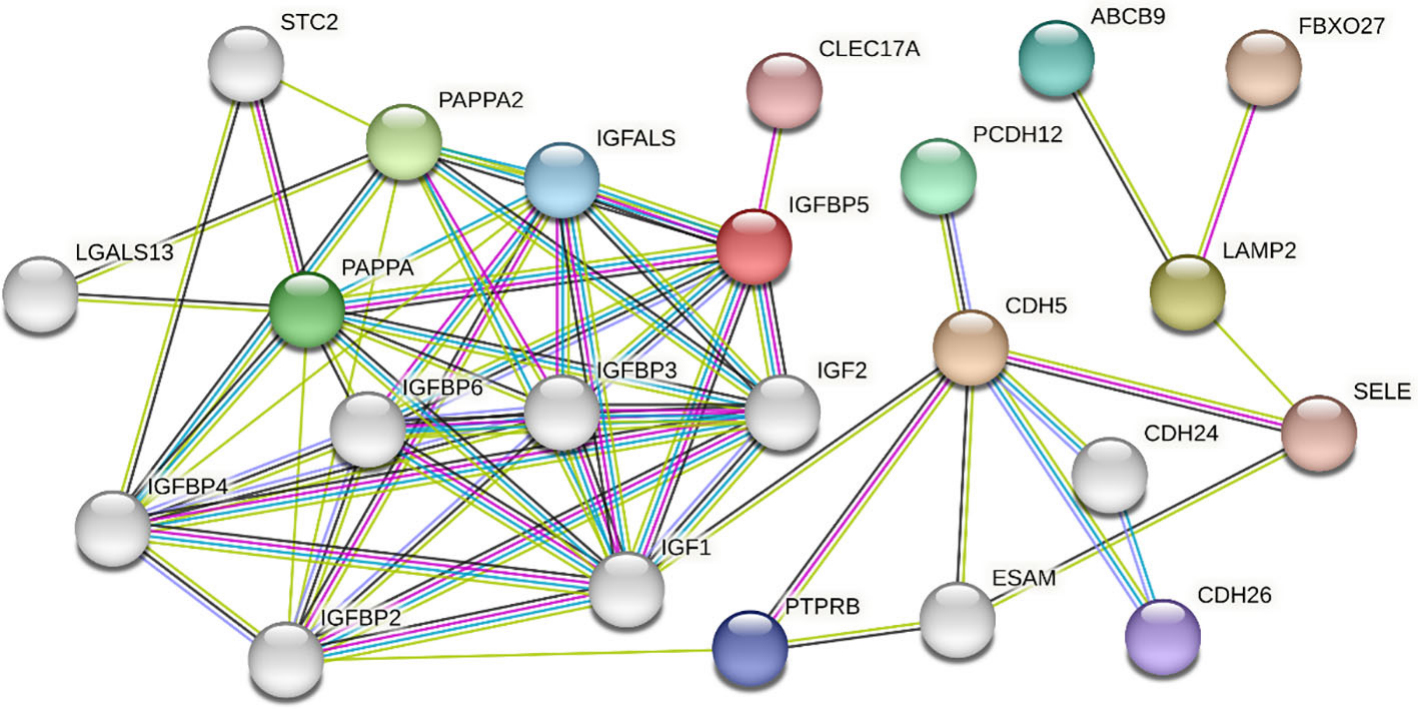
Analysis of direct and indirect interactions between three proteins based on information from the STRING database.

Finding direct and indirect interactions between target proteins (LAMP2, IGFBP5, and CDH5) based on information in the STRING database. The interacting protein networks of the three target proteins were identified using the STRING database and the networks were visualized (**Figure 4**). 20 interactors were identified in the graph. There were no direct interactions between the three proteins as shown in **Figure 4**. ABCB9, FBXO27 and SELE were found to interact directly with LAMP2. PCDH12, SELE, CDH24, CDH26, IGF1, PTPRB, and ESAM were found to interact directly with CDH5. CLEC17A, IGF2, IGF1, IGFBP3, IGFALS, IGFBP6, PAPPA2, and PAPPA are found to interact directly with *IGFBP5*. Other indirectly interacting proteins include STC2, LGALS13, IGFBP4 and IGFBP2.

## 4 Discussion

“Heterogeneity” is a highly distinctive feature of PCOS and inevitably contributes to its onset and progression. Therefore, it is necessary to characterize the changes in oocyte quality in PCOS and the mechanisms influencing them according to the type of PCOS. Previously, limited studies have focused on first categorizing patients by type before investigating the relationship between PCOS and embryonic outcomes, yet the findings of these studies have generally been consistent. For instance, patients with PCOS with phenotype A (coexistence of hyperandrogenism, ovulation disorders, and polycystic ovaries) or those with a combination of phenotypes, such as obesity, insulin resistance (IR), and metabolic syndrome, are at a significantly increased risk for decreased oocyte quality (1, 5).

The findings of the present study align with those of previous studies because, among the four phenotypes of PCOS, patients with phenotype D (i.e., uncomplicated hyperandrogenic status) have the lowest risk and degree of metabolic abnormalities (11). A study by Huang et al. concluded hyperandrogenism is a key factor contributing to impaired IR and other metabolic conditions (12). Consistent with the results of this study, patients in the hyperandrogenic PCOS group in the present study showed a significantly impaired glucose and lipid metabolism compared with those in the non-hyperandrogenic PCOS and control groups. Therefore, hyperandrogenic status may be one of the reasons for the decreased oocyte quality in patients with hyperandrogenic PCOS. The results from a few previous studies support this conclusion (5, 13–15). Statistical analysis of certain clinical data revealed that hyperandrogenism can affect the quality of oocytes in patients with PCOS, thereby significantly reducing the fertilization rate, rate of formation of high-quality embryos, rate of blastocyst formation, and rate of clinical pregnancy (5). In addition, animal studies have demonstrated that excess androgens can affect the quality of oocytes (13–15).

For the first time, protein profiles of FF in patients with hyperandrogenic PCOS were compared to those with non-hyperandrogenic PCOS and age- and BMI-matched general control patients using 4D label-free relative quantitative proteomics.

The FF protein profiles of hyperandrogenic PCOS patients were compared with those of control patients. The findings indicated that the proteins expressed differentially were primarily associated with immunity, inflammation and metabolism. Serum amyloid A-1 protein (SAA1), a marker of inflammation produced mainly by the liver, is significantly increased in peripheral blood, granulosa cells, FF, and endometrium of patients with PCOS, and its elevated expression can promote the occurrence of IR and miscarriage in PCOS patients (16, 17). Özdemir Başer and colleagues reported a significant increase in serum cystatin C levels among PCOS patients, which correlated positively with interleukin 6, an inflammatory factor (18). Additionally, matrix metalloproteinases (MMPs), which influence ovarian tissue remodeling and are involved in PCOS pathogenesis (19), were found to be elevated in PCOS patients, with higher serum levels of MMP9 and MMP2 leading to follicular dysplasia (20).

The insulin-like growth factor-binding proteins (IGFBPs) family modulates the bioavailability of insulin and insulin-like growth factor (IGF) through binding, thereby facilitating their respective physiological functions (21). Prior research has indicated that both IGFBP7 and IGFBP5 have significant roles in the progression of type 2 diabetes and its related complications (22, 23). Lysosome-associated membrane glycoprotein 2 (LAMP2), an autophagy-linked protein, has been linked to the development of various inflammatory diseases due to its altered expression. Notably, autophagy levels are markedly increased in ovarian tissues and granulosa cells of patients with PCOS or in PCOS model rats (24, 25). Lipopolysaccharide (LPS) stimulates the release of inflammatory factors, and its key ligand, lipopolysaccharide-binding protein (LBP), activates the inflammatory response by interacting with LPS. Studies have shown that LBP levels are significantly elevated in the serum of PCOS patients and correlate independently with IR in this condition (26). High endothelial permeability is pivotal in inflammatory and immune responses, and CDH5 serves as a crucial marker of endothelial permeability; lower CDH5 expression levels correspond to higher endothelial permeability (27).

Apolipoprotein E (ApoE) plays a crucial role in lipid clearance, and its dysregulation can contribute to the development of atherosclerosis and cardiovascular disease. In experimental studies of atherosclerosis, ApoE-/- mice are among the most commonly used transgenic models (28). Phospholipid transfer protein (PLTP), a key player in lipoprotein conversion and metabolism, has been positively linked to the risk of cardiovascular diseases, including atherosclerosis (29). Nevertheless, studies have revealed that PLTP deficiency can worsen glucose intolerance and inflammatory conditions induced by a high-fat diet (30). It’s noteworthy that plasma PLTP activity can be diminished by insulin infusion or hyperinsulinaemia (31, 32). Remarkably, elevated circulating PLTP levels notably enhance glucose tolerance and insulin sensitivity (33). Our current investigation demonstrates a significantly reduced PLTP expression in the hyperandrogenic PCOS group compared to the control group.

The proteins expressed differentially in the comparison between the hyperandrogenic and non-hyperandrogenic groups were likewise linked to immunity, inflammation, and metabolism. This finding indicates that a significant androgen elevation may intensify immune, inflammatory, and metabolic disruptions in individuals with PCOS. Previous studies by Kumtornrut et al. and Quartier et al. have shown that the introduction of androgens elevates the gene expression of IGFBP5 (34, 35). Significant increases in autophagy-related markers have been observed in granulosa cells upon exposure to specific concentrations of dihydrotestosterone or testosterone propionate (36, 37). Furthermore, hypogonadal men undergoing testosterone replacement therapy have demonstrated notable elevations in the count of peripheral blood monocytes and dendritic cells, as well as enhanced LAMP2 expression (38). Although no prior research has explored the connection between hyperandrogenism and CDH5 expression, patients with hyperandrogenic PCOS face a notably elevated risk of ovarian hyperstimulation syndrome (OHSS) and exhibit significantly heightened vascular endothelial cell permeability during hyperovulation. This observation offers indirect corroboration for our study’s findings. Furthermore, previous studies have demonstrated that androgen excess can increase food intake and promote obesity by suppressing insulin and leptin signaling in the hypothalamus and upregulating the expression of pro-phagocytic genes (39). Mimecan, recently recognized as a novel satiety hormone (40), showed significantly reduced expression in the FF of the hyperandrogenic PCOS group.

Numerous preceding investigations align with the findings of our current study. Zhang and colleagues revealed an imbalance in immune and inflammatory responses within the FF of individuals with PCOS (41). Gaberšček et al. determined that PCOS is marked by persistent low-grade inflammation accompanied by autoimmune sequelae (42). Furthermore, Ascani et al. illustrated that elevated androgen levels can modify the frequency of B lymphocytes and elevate circulating immunoglobulin M titers (43).

By cross-referencing, our study pinpointed three proteins—IGFBP5, LAMP2, and CDH5—that are differentially expressed and may mediate the effects of hyperandrogenism on oocyte quality. We subsequently confirmed their expression levels in FF via ELISA, finding concordance with our proteomics data. Correlation analyses indicated that higher IGFBP5 and LAMP2 levels, coupled with lower CDH5 levels, correlate with reduced oocyte quality. These observations align with previous research (44–48).

Kulus M et al. demonstrated that IGFBP5 is primarily expressed in atretic follicles and serves as a marker of senescence in ovarian granulosa cells cultured *in vitro* (44). When IGFBP5 levels rise significantly in FF, the likelihood of oocytes developing into blastocysts within follicles notably diminishes (45). Furthermore, Peng et al. and Zhong et al. reported that increased IGFBP5a transcription levels are linked to the embryotoxic effects of antioxidants in zebrafish (46, 47). Exposure to aflatoxin B1, high temperature, and nonylphenol, all known to harm oocyte mass, leads to an elevation in LAMP2 expression (48–50). Previous research has rarely explored the association between CDH5 and oocyte or embryo development. Xu and colleagues found that, following *in vitro* maturation (IVM) of immature oocytes, the expression of CDH5 was notably elevated in the granulosa cells adjacent to oocytes capable of developing into blastocysts, compared to those unable to progress to this stage (51).

Although so many studies have shown a correlation between alterations in the three and oocyte quality, the exact mechanism of action is not currently elucidated in detail. The results of protein interactions analyses showed no direct interactions among the three proteins. Further analysis of their indirect interactions revealed that the three proteins may work together to influence oocyte quality through co-regulation of cell proliferation, inflammatory response and other processes. The specific mechanism will be determined by further studies in the future.

In summary, IGFBP5, LAMP2, and CDH5 may be involved in the process by which hyperandrogenism affects oocyte quality in PCOS patients. In future studies, we plan to further explore these differential proteins to reveal the pathogenesis of hyperandrogenic PCOS and provide new ideas to improve oocyte quality of hyperandrogenic PCOS patients.

## Data Availability

The datasets presented in this study can be found in online repositories. The names of the repository/repositories and accession number(s) can be found below: http://www.proteomexchange.org/, PXD060939.
